# Supplementary material for: Leverage point themes within Dutch municipalities’ healthy weight approaches: A qualitative study from a systems perspective
Source: PLoS One. 2023 Jun 13;18(6):e0287050. doi: 10.1371/journal.pone.0287050 (PMC10263314; doi:10.1371/journal.pone.0287050)
Supplement: S1 Appendix — (DOCX) [file pone.0287050.s001.docx]

**S1 Appendix. Interview protocol in English**

**Remarks**

| Meaning of colors and symbols   - Orange: ASM model - Red: applies only to specific interviews (e.g., participants who work in multiple organizations or participants who participate together with old and new) - […]: name is filled in by researcher - Numbered questions: asked questions - : Possible in-depth question if answer was incomplete - a/b/c questions were always asked, unless the participant had already answered the question in a previous answer   Categories events  Physical living environment facilities   - This includes: things that are always present and visible in the public space that citizens can use (environmental design) that contribute to behavior that promotes healthy weight (such as nutrition, exercise, sleep, mental well-being, relaxation, poverty, etc.). It also includes schemes and resources such as financial aid - Examples are: cycle paths, supermarkets, sports facilities, QR fit routes in schools     Interventions and activities aimed at lifestyle   - This includes: individual or group meetings and programs, such as activities and interventions, that are organized by people and that focus on behaviors that promote healthy weight (such as nutrition, exercise, sleep, mental well-being, relaxation, poverty, etc.) - Examples: walking groups, combined lifestyle interventions, monthly sports offer, consultation with a dietician, buddy projects     Working groups   - This includes: collaborations between people and/or organizations that result in interventions, activities, or agreements that are at least partly aimed at, or result in, behavior that promotes healthy weight (such as nutrition, exercise, sleep, mental well-being, relaxation, poverty, etc.) - Examples: working group from the local prevention agreement, citizens’ initiative foundations     Healthy lifestyle woven into regular activities   - This includes: repeated (more than once) promotion aimed at behavior that promotes healthy weight (such as nutrition, exercise, sleep, mental well-being, relaxation, poverty, etc.) during daily work directly or indirectly aimed at a person or group - Examples: a general practitioner pointing out a healthy lifestyle to his/her patient, putting up Nutrition Center posters in the physiotherapist’s waiting room, working groups that consume fruit with coffee instead of biscuits |
| --- |

**Start interview protocol** - *Only part 1 is provided as part 2 does not relate to this paper*

**Introduction**

- *Can you see/hear me well?*
- *I am very happy that you want to join this conversation. Do you have any questions beforehand?*
- *The data from the conversation are processed anonymously. There are no right or wrong answers, just indicate how you experience things.*
- *Do you mind if I record the conversation?*

*Introduction text – In principle, one hour is scheduled for this conversation, but we notice that the conversations last one hour and a quarter; it also depends on how much and how enthusiastically you talk. Do you possibly have a little more time?*

*[Part 1+2] Introduction text – This conversation consists of two parts. In the first part, we want to talk about the healthy weight approach in [municipality]. This will cover most of our conversation. The second part is about the learning community.*

*[Part 1] Introduction text - We want to talk about the healthy weight approach in [municipality].*

*[If two participants] I can imagine that [new name] might have a little less to say about the topics. This conversation can also serve as a transfer. [new name] you could complete or interrupt [old name] if you have additions or other ideas.*

**PART 1: The system** (Nobles, 2021)

*Then we start with the first question.*

1. Would you like to describe in two sentences what your [function] in [municipality(s)] entails?

*[Track which categories are named]*

[If several municipalities] Today we are going to talk about one municipality in particular. A comparative question will sometimes be asked about the other municipality. Which municipality do you mainly want to talk about?

*Introduction text – During this conversation, we are talking about what you see in [municipality] that promotes or hinders healthy weight. Think, for example, of activities and facilities aimed at nutrition, mental well-being, and poverty. This is called the healthy weight approach. We are going to talk about what you see of the healthy weight approach.*

**Events within the healthy weight approach**

1. Can you briefly summarize which **activities** aimed at lifestyle you are involved in as [function] in [municipality]? *(Events)*
2. Are you involved in the **design and facilities** linked to lifestyle in [municipality]? **If yes, can you briefly list what?** *(Events)*
3. Can you briefly list which **working groups** you are involved in within the healthy weight approach in [municipality]? *(Events)*
4. Are there **other things** you do in your **work** to promote the lifestyle of others in [municipality]? Think, for example, of serving a healthy lunch or encouraging a healthy lifestyle. **If so, can you briefly summarize what you are involved in?** *(Events)*

*[If the researchers don’t have a clear overview]* If you sum it up now, can you list what you are involved in?

*[Track which categories are named]*

[If someone works for several municipalities: How is this different in [municipality]?
Now we go back to [municipality].

1. You mentioned that you are involved in *[category 1]. We are now going to talk about [category 1].* How are you involved in [category 1] in [municipality]? *(Structure)*
   1. Which organizations and functions do you work with?

*[Note organizations] (Structure)*

- 1. Why do you work with these organizations and functions? **Think for example about money, time, knowledge, or connections.** *(Structure)*

*[If not all organizations are listed]* Why are you working with [organization]? *(Structure)*

- - - Are there any other reasons for working with [these organizations and functions]? *(Structure)*
  1. What do you think is good or bad about the collaboration? *(Structure)*

*[If not identified]* Does this apply to all named organizations and functions? *(Structure)*

[If someone works for several municipalities: How is this different in [municipality]?
Now we go back to [municipality].

*[If two participants] [If the new person has not/barely spoken] - [name new] How do you see this? Do you have any additions or other ideas about this?*

*[Ask questions below for 2, 3, and 4]*

1. You mentioned that you are involved in *[category]. We are now going to talk about [category].* How are you involved in [category] in [municipality]? *(Structure)*
   1. Which **other** organizations and functions do you work with?

*[Note organizations] (Structure)*

- 1. *[Only for new organizations]* Why do you work with these organizations and functions? **Think for example about money, time, knowledge, or connections.** *(Structure)*

*[If not all organizations are listed]* Why are you working with [organization]? *(Structure)*

- - - Are there any other reasons for working with [these organizations and functions]? *(Structure)*
  1. *[Only for new organizations]* What do you think is good or bad about the collaboration? *(Structure)*

*[If not identified]* Does this apply to all named organizations and functions? *(Structure)*

[If someone works for several municipalities: How is this different in [municipality]?
Now we go back to [municipality].

*[If categories are mixed up]* I understand that [category] and [category] are related. If we are talking about [category] specifically. [Ask question again]

[Check that all categories have been discussed]

*[If two participants] [If the new person has not/barely spoken] - [name new] How do you see this? Do you have any additions or other ideas about this?*

*Introduction text – We talked about where you are involved in the healthy weight approach. We are now going to talk about what you see about the healthy weight approach. This includes everything you see in [municipality] that hinders or promotes healthy weight. Think for example of activities and facilities aimed at nutrition, mental well-being, and poverty.*

**Points of attention**

1. When we talk about what you see about the healthy weight approach. What do you think is going well? *(Events*)
   1. What do you mean? What does this action entail? *(Events*)
2. What are the biggest points of attention that you keep experiencing within the healthy weight approach? *(Events*)

What are the biggest points of attention that you keep experiencing related to the **impact and content** of the healthy weight approach? *(Events*)

What are the biggest points of attention that you keep experiencing related to the **organization** of the healthy weight approach? *(Events*)

What do you mean? What does this mean in practice? *(Events*)

- 1. Why are these points of attention? *(Structure, Goals)*

[If someone works for several municipalities: How is this different in [municipality]?

Now we go back to [municipality].

*[If two participants] [If the new person has not/barely spoken] - [name new] How do you see this? Do you have any additions or other ideas about this?*

**Solutions***You have indicated a number of points of attention. Now we are looking for solutions.*

*[Go through the following questions for each major point of attention]*

*When we talk about [point of attention]*

1. What actions would partially resolve [point of attention] in the future? *[This is about what, not who]* (*Events*)

*[If the participant mentions that others have to work on this]* What actions could [named party] perform in the future? (*Events; Structure*)

What do you mean? What does this action entail? What does this mean in practice? (*Events*; *Structure*)

- 1. What of this is already being done to resolve [point of attention]? (*Events*)

1. Which organizations in [municipality] should take responsibility for [point of attention]? *(Structure, Beliefs)*

*[If outside the municipality]* Which organizations can take responsibility within [municipality]? *(Structure, Beliefs)*

- 1. How does this fit with the goal of this organization? *(Goals, Beliefs)*

1. If you started working with [point of attention], what would your work environment (such as manager) think of this? *(Beliefs)*

*[If participant has already started working on it]* What does your work environment (such as manager) think about that you are working with [point of attention]? *(Beliefs)*

- 1. Does this fit within the goals of your organization? *(Goals)*
  2. *[If no]* Is this a barrier to getting started? *(Beliefs)*

[If someone works for several municipalities: How is this different in [municipality]?
Now we go back to [municipality].

[Check that all points of attention are discussed]

1. From what we have just discussed, what change can **you** initiate first? *(Events)*

How can this be organized? *(Structure)*

Who are involved? *(Structure)*

*[If two participants] [If the new person has not/barely spoken] - [name new] How do you see this? Do you have any additions or other ideas about this?*

*Introduction text – We now move to the last part of the first part of the conversation. This is about your goal within the healthy weight approach.*

**Goals**

1. If you look from yourself as [function]. Suppose that with your efforts you have achieved everything you wanted to achieve in [municipality], what does [municipality] look like? What has changed compared to now? *(Goals)*

Why do you think this goal is important from your position as [function]? *(Beliefs)*

- 1. What are you doing to achieve this goal? *(Events)*
     1. What does this look like in practice?

1. [Go through this question for every individual/organization the participant works with]

You indicated that you work with [person/organization]. If we are talking about the entire organization, what is the ultimate goal of [person/organization]? *(Goals)*

What goal are you working on together? *(Structure, Goals)*

Where do your goals differ? These can also be goals outside the healthy weight approach. *(Goals)*

- 1. How important do you think it is to [person/organization] to strengthen the healthy weight approach? *(Beliefs)*

1. Are there any other important things about the healthy weight approach in your municipality that we did not discuss yet?

*[If two participants] [If the new person has not/barely spoken] - [name new] How do you see this? Do you have any additions or other ideas about this?*
